# Whole‐Brain 4D MRA With Improved Vessel Delineation Using Undersampled Multiple Overlapping Thin‐Slab Radial Acquisition

**DOI:** 10.1002/nbm.70426

**Published:** 2026-10-22

**Authors:** Tianrui Zhao, Sang Hun Chung, Yining He, Ziyi Huang, Jianing Tang, Sameer A. Ansari, Lirong Yan

**Affiliations:** ^1^ Department of Radiology Feinberg School of Medicine, Northwestern University Chicago Illinois USA; ^2^ Department of Biomedical Engineering Northwestern University Evanston Illinois USA

**Keywords:** 4D MRA, arterial spin labeling (ASL), golden‐angle radial, multiple overlapping thin‐slab acquisition (MOTSA), noncontrast‐enhanced MRA

## Abstract

Time‐resolved arterial spin labeling (ASL)‐based noncontrast 4D MRA enables visualization of cerebral arterial flow dynamic patterns and holds great potential for evaluating cerebrovascular disease. However, conventional ASL 4D MRA implementations using look‐locker acquisitions are susceptible to progressive RF saturation during extended readouts, leading to substantial ASL signal attenuation and loss of vessel delineation in distal arteries. These limitations potentially hinder a wider clinical translation of 4D MRA, especially when assessment of whole‐brain vascular anatomy and flow dynamics is needed. This study introduces a practical and reliable ASL 4D MRA technique for whole‐brain or large volumetric imaging by integrating pulsed ASL with undersampled multiple overlapping thin‐slab stack‐of‐stars (SOS) golden‐angle radial acquisition. The proposed method was evaluated against conventional single‐slab 4D MRA, 3D time‐of‐flight magnetic resonance angiography (TOF‐MRA), and pseudocontinuous ASL (pCASL)‐based 4D MRA in terms of vessel delineation, blood flow dynamics, and image quality. Compared to the other methods, the proposed method substantially reduced RF saturation effects and preserved signal intensities in distal small arteries, resulting in significantly improved vessel continuity and contrast throughout the cerebral vasculature, as well as reliably capturing complete inflow‐to‐outflow arterial flow dynamics with good scan efficiency and image quality. The proposed technique enables high‐quality, time‐resolved whole‐brain angiography, offering a promising noncontrast imaging tool for comprehensive assessment of cerebral arterial hemodynamics in clinical and research applications.

## Graphical abstract

A noncontrast‐enhanced 4D MRA technique by integrating pulsed ASL with undersampled multiple overlapping thin‐slab stack‐of‐stars golden‐angle radial acquisition is developed for the evaluation of whole‐brain arterial flow dynamics with improved vessel delineation. The proposed method substantially reduced RF saturation effects and preserved signals in distal arteries, providing improved visualization of whole‐brain vasculature and arterial dynamics with good image quality and scan efficiency.

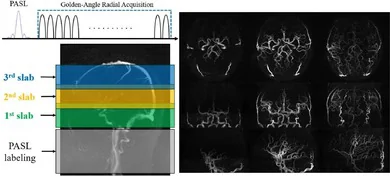

**Abbreviations**
3D: three dimensional
4D: four dimensional
ASL: arterial spin labeling
ce
: contrast‐enhanced
cMIP: collapsed maximum intensity projection
CNR: contrast‐to‐noise ratio
FA: flip angle
FFT: fast Fourier transform
FOV: field of view
GRAPPA: generalized autocalibrating partially parallel acquisitions
MIP: maximum intensity projection
MOTSA: multiple overlapped thin‐slab acquisition
MRA: magnetic resonance angiography
PASL: pulsed arterial spin labeling
pCASL: pseudocontinuous arterial spin labeling
RF: radio frequency
SNR: signal‐to‐noise ratio
SOS: stack of stars
SS: single slab
STAR: signal targeting with alternative radio frequency
TOF: time of flight
VFA: variable flip angle

## 1 Introduction

Cerebrovascular diseases are a leading cause of disability and mortality worldwide. The comprehensive characterization of cerebral blood flow hemodynamics is essential for accurate clinical diagnosis, treatment planning, and disease monitoring [1, 2, 3]. Digital subtraction angiography remains the gold standard in clinical practice for assessing cerebrovascular anatomy and flow dynamics due to its high spatial and temporal resolution [4]; however, its invasive profile and associated ionizing radiation limit its suitability for routine or longitudinal use. Magnetic resonance angiography (MRA) has emerged as an important noninvasive modality and has become widely adopted in clinical routine [5]. Among MRA techniques, 3D time‐of‐flight (TOF) MRA is the mainstay for evaluating cerebrovascular abnormalities because of its high spatial resolution [6, 7]. Nevertheless, TOF‐MRA is inherently limited by its inability to provide flow dynamic information, which is critical for the assessment of various cerebrovascular disorders, such as arteriovenous shunting in arteriovenous malformations (AVM) and collateral circulation in stenosis‐occlusive diseases [8, 9, 10, 11, 12]. Time‐resolved contrast‐enhanced (ce) MRA can depict flow dynamics [13, 14], but its performance is constrained by an inherent trade‐off between spatial and temporal resolution due to the need to capture the first‐pass contrast bolus within a limited acquisition window, restricting its ability to adequately resolve temporal dynamics. Arterial spin labeling (ASL), as a noninvasive MRI technique, was originally developed for perfusion imaging by using magnetically labeled arterial blood as an endogenous tracer [15, 16]. It can be naturally extended to produce angiographic contrast when labeled blood spins reach cerebral arteries with a short or no post‐labeling delay following the labeling preparation [15, 17]. Recent advances in fast imaging and reconstruction methods have facilitated the development of pulsed ASL (PASL)‐based time‐resolved four‐dimensional MRA (4D MRA), particularly through radial sampling strategies combined with advanced image reconstruction [18, 19, 20, 21, 22, 23]. These developments have substantially improved the feasibility of capturing dynamic vascular information within short acquisition times without compromising spatiotemporal resolution, thereby enhancing the clinical translation of PASL‐based 4D MRA for evaluating cerebrovascular hemodynamics [24, 25, 26].

Despite these promising advancements, technical challenges remain in PASL‐based 4D MRA. Look‐locker acquisition with repeated fast gradient‐echo readout after each ASL preparation is typically employed in 4D MRA to capture the full passage of labeled blood flow through the cerebral vasculature. However, blood T1 relaxation and repeated RF excitations during the long readout train often lead to substantial ASL signal attenuation, particularly with extensive spatial coverage, e.g., whole brain. This effect becomes pronounced in distal small arteries and/or vascular lesions with abnormally slow flows. As a result, most existing PASL‐based 4D MRA implementations employ single‐slab acquisition with relatively narrow spatial coverage, e.g., the circle of Willis and its major branches [18, 19, 20, 21, 22]. Such limited spatial coverage may restrict their applicability in clinical scenarios that require whole‐brain vascular assessment, such as large AVM lesions with complex venous drainage and moyamoya disease with extensive collateral circulation. Although pseudocontinuous ASL (pCASL), as an alternative labeling strategy in 4D MRA, enables whole‐brain MRA with good delineations of both proximal and distal cerebral arteries [27, 28], it inevitably increases acquisition time with the same acquisition readout as PASL 4D MRA because of the longer labeling duration. It also results in signal evolution that is more strongly influenced by blood outflow during image acquisition. Consequently, depiction of inflow dynamics may be less direct than in PASL‐based 4D MRA and often requires additional post‐processing approaches to approximate inflow information.

In this study, we propose a practical and robust whole‐brain PASL‐based 4D MRA technique that improves distal vessel delineation while maintaining high spatiotemporal resolution within a clinically reasonable scan time. The proposed method combines stack‐of‐stars (SOS) golden‐angle radial sampling with an undersampled multiple overlapping thin‐slab acquisition (MOTSA) strategy, analogous to the approach used in 3D TOF‐MRA to mitigate inflow signal attenuations and enhance blood contrast, especially for slow or distal blood flows. Through in vivo experiments, we evaluated the feasibility and performance of the proposed technique by comparing it with a conventional single‐slab 4D MRA with matched spatial coverage and resolution. Further comparisons were made against 3D TOF‐MRA and pCASL‐based 4D MRA in terms of vessel delineation, depiction of blood flow dynamics, and image quality.

## 2 Methods

### 2.1 Pulse Sequence Design

The schematic of the proposed technique is illustrated in Figure 1. A pulsed ASL preparation using the signal targeting with the alternative radio frequency (STAR) labeling scheme was employed to tag arterial blood spins inferior to the imaging volume. The PASL labeling volume was fixed below the bottom imaging slab and remained unchanged across all imaging slabs to ensure consistent labeling conditions and blood dynamics. The labeling module was immediately followed by a spoiled gradient‐echo readout with SOS golden‐angle (111.246°) radial sampling for data acquisition [29] (Figure 1a). Before each magnetization preparation, a spatially selective presaturation pulse covering the imaging volume was applied to null residual signals and suppress background tissue signals. Label and control acquisitions were interleaved per partition (kz) encoding. For each kz encoding, all in‐plane radial spokes were acquired within a single TR (single‐shot acquisition) before proceeding to the next partition encoding, enabling capture of the complete inflow‐to‐outflow dynamics of the labeled bolus.

**FIGURE 1:**
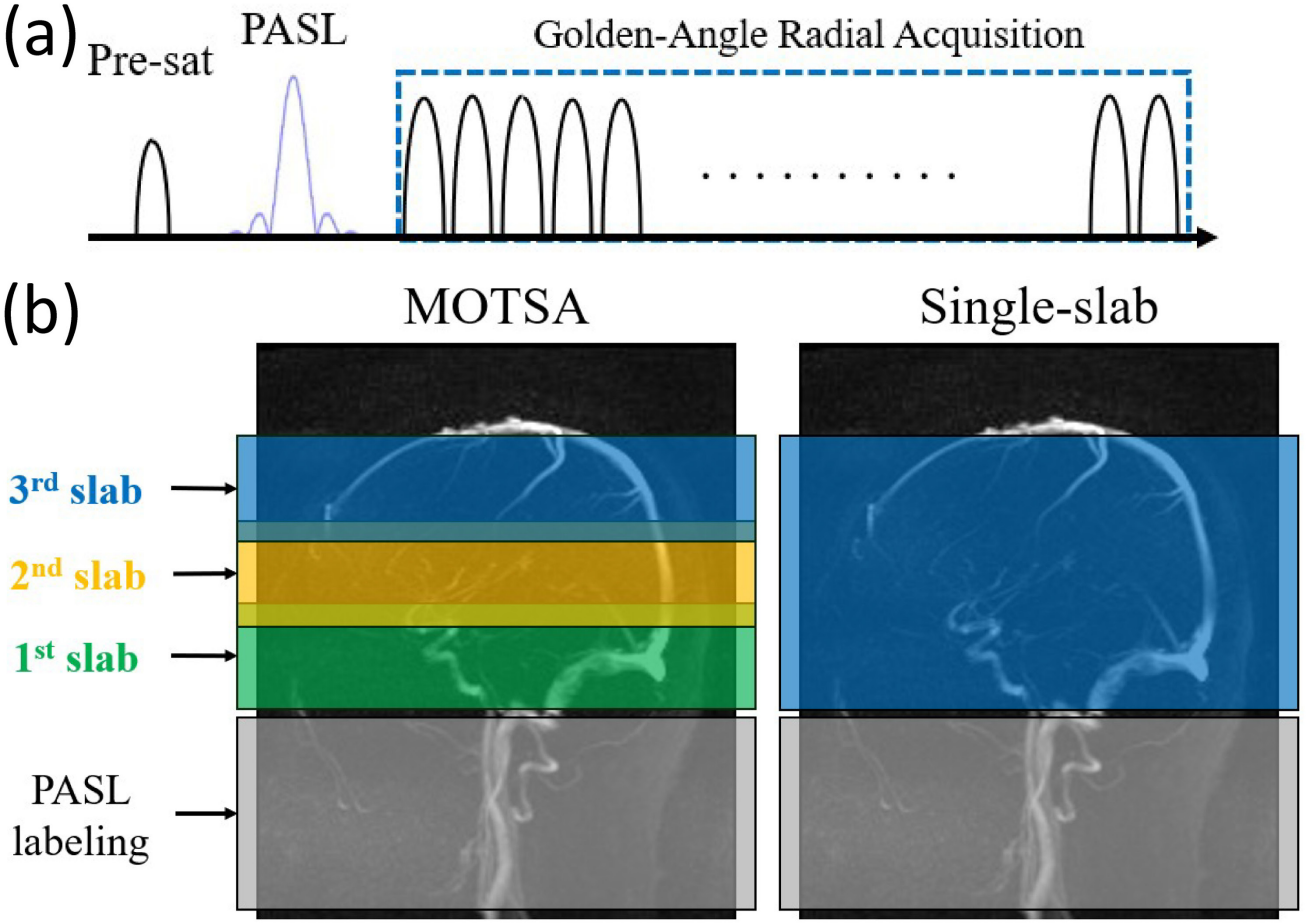
(a) Pulse sequence diagram. A pulsed ASL was used for magnetization preparation, followed by a stack‐of‐stars golden‐angle radial spoiled gradient‐echo readout. A presaturation pulse was applied prior to each labeling module to suppress the background tissue signal. (b) Illustration of the imaging scheme. Compared to conventional single‐slab acquisition, the multiple overlapping thin‐slab acquisition strategy, in which three partially overlapping slabs were acquired sequentially in an ascending order along the superior–inferior direction to reduce RF saturation effects and preserve the distal arterial signal.

To improve spatial coverage and mitigate RF‐induced signal saturation effects, MOTSA was implemented. Specifically, compared to the conventional 4D MRA with single‐slab acquisition, three partially overlapping thin slabs were acquired sequentially, and only one of the narrow imaging slabs was excited per magnetization preparation (Figure 1b). This approach reduces cumulative RF saturation experienced by inflowing labeled blood as it travels through the entire imaging volume, thereby preserving ASL signal and improving vessel delineation in distal arteries and regions with slower flow. To enhance the scan efficiency, linear undersampling was applied along the partition direction, with an undersampling factor of 3 in the peripheral partitions. Specifically, all central kz partitions were fully sampled, whereas kz partitions in peripheral regions were linearly undersampled (skipped) during acquisition.

### 2.2 MRI Experiment

Seven healthy participants (7 males, 25 ± 3.46 years) were enrolled in this study after approval from the local institutional review board, and written informed consent was obtained from all participants. All MRI scans were conducted on a Siemens 3 T MRI system (Prisma, Siemens Healthcare, Erlangen, Germany) using a 64‐channel head/neck coil.

The imaging parameters of the proposed sequence (abbreviated as MOTSA MRA) included: field of view (FOV) = 208 × 208 mm^2^; voxel size = 1 × 1 × 1 mm^3^; three overlapping slabs, each comprising 48 slices and 16.7% slice oversampling (8 slices, 8 mm); slab distance factor = −15%. SOS sampling included 20 fully sampled central calibration partitions for each slab. The flip angle (FA) = 8°; bandwidth = 200 Hz/pixel; TE/echo spacing = 3.31/7.5 msec. For each partition encoding, 300 in‐plane radial spokes were acquired in a single shot. The total scan time was 7 min 22 s.

For comparison, a conventional single‐slab 4D MRA (abbreviated as SS MRA) with matched spatial coverage and imaging parameters was collected, except that a single slab comprising 128 slices and 12.5% slice oversampling (16 slices, 16 mm) was used. The number of fully sampled central calibration partitions was 66. The total scan time was 7 min 4 s.

A high‐resolution whole‐brain 3D TOF‐MRA sequence with the same spatial coverage was also performed with imaging parameters: FOV = 208 × 208 mm^2^; acquired voxel size = 0.8 × 0.8 × 0.6 mm^3^ with 2× interpolation, resulting in a reconstructed voxel size = 0.4 × 0.4 × 0.6 mm^3^; seven slabs with each comprising 36 slices and 22.2% slice oversampling (8 slices, 8 mm); slab distance factor = −16.67%; FA = 18°; bandwidth = 186 Hz/pixel; TE/TR = 3.5/21 msec. The total scan time was 11 min 16 s.

To comprehensively evaluate vascular delineation and flow dynamics, a pCASL‐based 4D MRA sequence using the same readout (abbreviated as pCASL MRA) was acquired with identical spatial coverage and imaging parameters matched to the SS MRA, except for labeling duration = 1 s. The total scan time was 10 min 8 s. In addition, a single thin‐slab pCASL‐based 4D MRA (abbreviated as thin‐pCASL MRA) with parameters matched to the top slab of MOTSA MRA (Figure 1b) was performed to enhance the visualization of distal arteries with a total scan time of 3 min 32 s. No post‐labeling delay was applied in either pCASL‐based 4D MRA sequence.

### 2.3 Image Reconstruction

All collected raw k‐space data from MOTSA MRA, SS MRA, pCASL MRA, and thin‐pCASL MRA were transferred to an external server (Linux 7; CPU: Intel Xeon Gold 6338 2GHz; GPU: NVIDIA RTX A6000; RAM: 192 GB) for offline reconstruction using in‐house MATLAB programs (MathWorks, USA).

For all datasets, GRAPPA was first applied along the kz direction to restore the undersampled partitions [30]. Postprocessed datasets were subsequently compressed into eight virtual coil channels to reduce computational complexity. The fast Fourier transform (FFT) was performed along the kz direction, enabling slice‐by‐slice image reconstruction; 300 total radial spokes per slice were retrospectively binned into 10 spokes per frame, resulting in a temporal resolution of 75 msec/frame (corresponding to an in‐plane acceleration rate of 32.7).

Image reconstruction was performed using a previously developed self‐calibrated low‐rank subspace reconstruction method [23, 31]. Briefly, the temporal basis representing signal dynamics was estimated from the k‐space center point using principal component analysis (PCA), which provides a compact low‐rank representation of the dominant temporal signal variations while suppressing noise contributions. The resulting temporal subspace model enabled reconstruction in a lower‐dimensional feature space, thereby improving computational efficiency and reducing memory requirements. For MOTSA MRA, a same temporal subspace model was derived jointly from data across all slabs to ensure consistency of image contrast and dynamic signal range throughout the reconstructed volume. Given the differing temporal behaviors of static tissue in PASL‐based and pCASL‐based 4D MRA, only the subtraction‐based temporal basis was used in the low‐rank model to reduce potential confounding effects arising from differences in tissue signal evolution. The regularization parameters ($\lambda_{1}=0.03,\;\lambda_{2}=0.005,\lambda_{3}=0.0001$) were empirically selected based on overall image quality and temporal fidelity, as described in the previous work. Label and control images were reconstructed jointly and subsequently subtracted to generate the dynamic angiographic image series. For visualization of blood flow dynamics, maximum intensity projections (MIPs) were generated from the dynamic angiograms in the transverse, coronal, and sagittal orientations. Collapsed MIP (cMIP) images were generated by applying an additional MIP across the temporal dimension of the dynamic MIP series. For MOTSA MRA, adjacent slabs were combined by averaging the slices within the overlapping regions (7 overlapping slices corresponding to a slab distance factor of −15%) to reduce slab‐boundary discontinuities. An example of the slab‐combination procedure is provided in Figure S1.

Because pCASL‐based MRA primarily depicts blood outflow dynamics due to a relatively long labeling duration before the readout, a subtraction‐based post‐processing approach was applied to approximate inflow dynamics [32]. Specifically, the first MIP frame was subtracted from the MIP of each subsequent frame to generate pseudoinflow MIP images. The resulting images were intensity‐inverted, and negative pixel values were removed to enhance visualization of inflowing blood signals.

### 2.4 Image Evaluation

#### 2.4.1 Blood Flow Dynamics

To evaluate blood dynamics, seven arterial regions of interest (ROIs) were manually drawn on the MIP images including: internal carotid artery (ICA); basilar artery (BA); M2/3 and M4 segments in middle cerebral artery (MCA); P1/2 and P3/4 segments in posterior cerebral artery (PCA); A1/2 segment in anterior cerebral artery (ACA). Each ROI consisted of 28 pixels and was placed consistently across subjects and imaging techniques. Dynamic signal intensity curves were generated by averaging the signals within each ROI for every temporal frame.

For comparison between MOTSA MRA and SS MRA, dynamic curves were normalized to the maximum ICA signal intensity within each respective dataset. A signal saturation factor was defined for each ROI as the ratio of the maximum dynamic signal intensity in SS MRA to that in MOTSA MRA, providing a quantitative measure of saturation‐related signal loss.

For comparison between MOTSA MRA and pCASL MRA, dynamic curves were normalized to the maximum signal intensity within each ROI and each technique independently. Because pCASL MRA primarily depicts blood outflow dynamics, the corresponding dynamic curves were temporally shifted to align their peak with those of the MOTSA MRA curves to facilitate qualitative comparison. Dynamic curves from all ROIs were averaged across subjects.

#### 2.4.2 Image Quality and Contrast

To quantitatively evaluate the image quality and contrast, the signal‐to‐noise ratio (SNR), contrast‐to‐noise ratio (CNR), and SNR efficiency were calculated for each of the seven ROIs. A 15 × 15 pixel region located in the upper‐left corner of the image was used to estimate background noise for SNR calculations, whereas a 15 × 15 pixel region placed within normal‐appearing brain tissue served as the reference for CNR calculations. To facilitate comparison of proximal‐to‐distal vessel behavior, the ROIs were grouped into three vessel levels: Level 1 (ICA and BA), Level 2 (M2/3, P1/2, and A1/2), and Level 3 (M4 and P3/4). SNR, CNR, and SNR efficiency values were averaged across subjects and vessel levels and are reported as mean ± standard deviation. To evaluate the robustness of the quantitative findings with respect to the reconstruction framework, all 4D MRA datasets were additionally reconstructed using a nonuniform fast Fourier transform (NUFFT) reconstruction [33]. SNR, CNR, and SNR efficiency were then evaluated using the same procedure described above.

#### 2.4.3 Vessel Delineation

Vessel delineation was quantitatively evaluated through vessel segmentation generated by a previously developed 3D spatial deep learning‐based neural network [34], which was applied separately to the MOTSA MRA, SS MRA, and pCASL MRA datasets. To minimize the influence of isolated segmentation artifacts, vessel skeletons were extracted from the binarized vascular masks, and small disconnected skeleton components were removed using connectivity‐based postprocessing. The total skeleton length was computed for each dataset and averaged across subjects, with results reported as mean ± standard deviation. To further validate the vessel delineation analysis, manual vessel segmentations were independently performed by two raters, with images randomly shuffled and blinded to the raters. The vessel skeleton length was computed for each manual segmentation and subsequently averaged between raters.

#### 2.4.4 Statistical Analysis

Statistical comparisons among the MOTSA MRA, SS MRA, and pCASL MRA techniques were performed using repeated measures analysis of variance (ANOVA), followed by post hoc pairwise comparisons. Differences in SNR, CNR, SNR efficiency, and vessel skeleton length were considered statistically significant at a two‐tailed *p*‐value < 0.05. Interrater agreement of vessel skeleton length in manual segmentation was evaluated through Bland–Altman analysis [35] and intraclass correlation (ICC) coefficient based on a two‐way random effects model with absolute agreement [ICC (1, 2)] [36].

## 3 Results

Figure 2 presents axial dynamic MIP images and cMIP images from a representative subject comparing MOTSA MRA and SS MRA. Both techniques demonstrated similar overall arterial inflow‐to‐outflow temporal patterns. However, SS MRA exhibited pronounced blood signal decay, particularly in distal arteries, resulting in reduced signal intensities and diminished vessel contrast compared with MOTSA MRA (red arrows).

**FIGURE 2:**
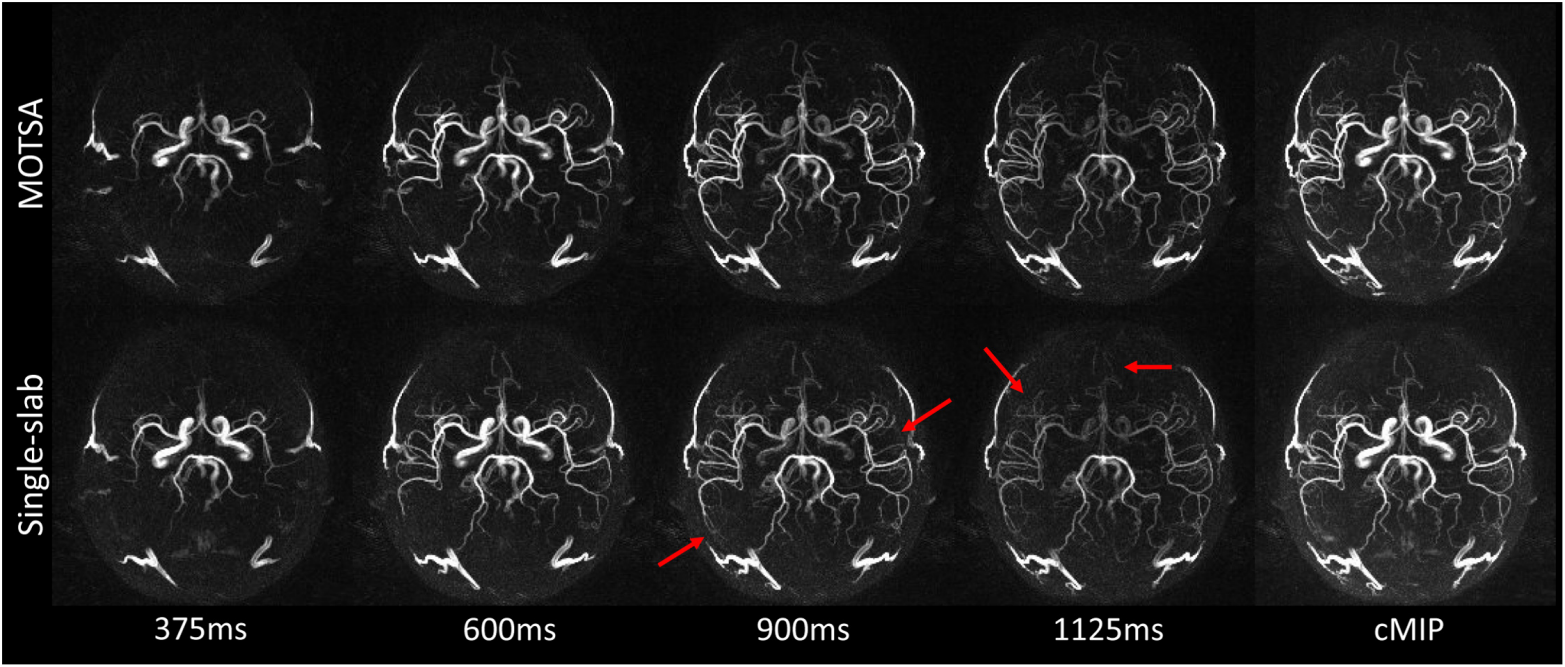
Representative axial dynamic MIP and cMIP images comparing the proposed MOTSA MRA and SS MRA. Both techniques show similar overall inflow‐to‐outflow dynamics, whereas SS MRA exhibits progressive signal attenuation and reduced vessel contrast in distal arteries (red arrows) due to RF saturation.

These observations were further supported by the quantitative blood dynamic curves shown in Figure 3. Across all seven arterial ROIs, signal intensity was consistently lower in SS MRA than in MOTSA MRA. The signal reduction increased progressively from proximal to distal arterial segments, reflecting cumulative RF saturation effects during blood transit. This trend is quantitatively evaluated by the signal saturation factor reported in Table S1, which demonstrates increasing saturation from major arteries to distal branches.

**FIGURE 3:**
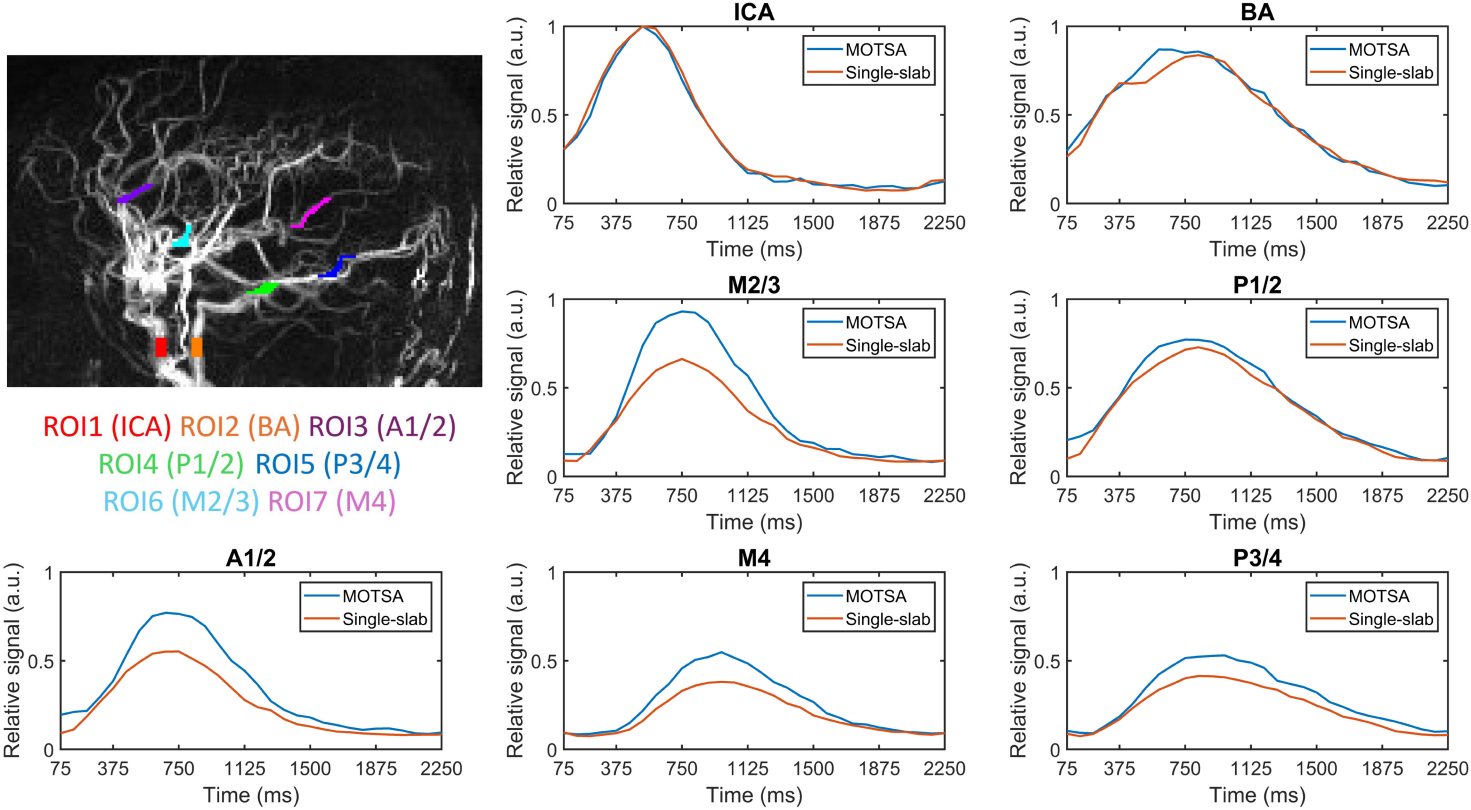
Dynamic signal intensity curves measured in seven arterial ROIs for MOTSA MRA and SS MRA. Signal intensity is consistently reduced in the single‐slab acquisition, with increasing attenuation from proximal to distal arterial segments, reflecting cumulative RF saturation effects.

Figure 4 compares sagittal cMIP and MIP images from four representative subjects acquired using MOTSA MRA, SS MRA, and TOF‐MRA. MOTSA MRA provided continuous visualization of the cerebral arterial tree with improved visualization of both proximal and distal branches compared with SS MRA and overall vessel depiction approaching that observed with TOF‐MRA. In contrast, SS MRA showed loss of fine vascular details, particularly in distal arteries, due to stronger signal attenuation (red arrows). Compared to TOF‐MRA, ASL‐based 4D MRA inherently suppressed background tissue signal through label/control subtraction, resulting in reduced background tissue signal as well as no venous signal contamination (yellow arrows). In addition, some thin vessels present in TOF‐MRA were not obvious in ASL‐based MRA, presumably due to the intrinsic high spatial resolution used in TOF‐MRA. Mild Venetian blind artifacts were observed in some MOTSA MRA datasets, consistent with the slab‐based acquisition strategy and similar to those observed in TOF‐MRA.

**FIGURE 4:**
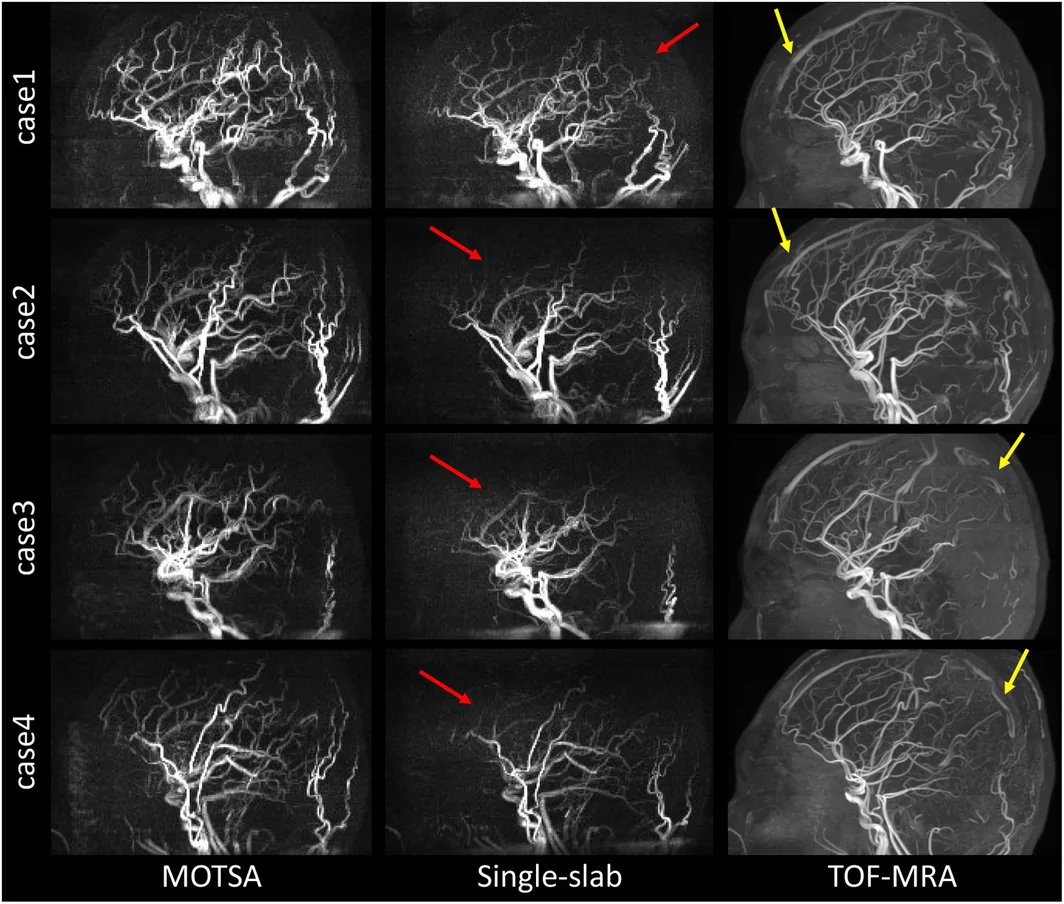
Sagittal cMIP and MIP images from four representative subjects acquired from MOTSA MRA, SS MRA, and TOF‐MRA. MOTSA MRA provides continuous visualization of distal arterial branches comparable to TOF‐MRA, whereas SS MRA shows loss of fine vessel detail (red arrows). ASL‐based 4D MRA also demonstrates effective background suppression and no venous signal contamination (yellow arrows). Mild Venetian blind artifacts were observed in some MOTSA MRA cases, similar to TOF‐MRA.

Figure 5 compares cMIP images from the top slab of MOTSA MRA, thin‐pCASL MRA, and the corresponding subregion extracted from SS MRA in three orthogonal views. MOTSA MRA substantially improved distal arterial delineation relative to SS MRA (red arrows) and achieved vessel visualization approaching that of thin‐pCASL MRA. Notably, MOTSA 4D MRA achieved this performance with two‐thirds of the acquisition time of pCASL‐based MRA for equivalent spatial coverage (2 min 21 s vs. 3 min 32 s). In some cases, thin‐pCASL MRA also presents stronger distal vessel delineation and contrast. This observation is likely related to the narrow imaging slab and short distance between the labeling plane and imaging slab in the thin‐slab pCASL acquisition, resulting in reduced signal decay and RF saturation. Corresponding comparisons with single large slab pCASL acquisition are shown in Figure S2.

**FIGURE 5:**
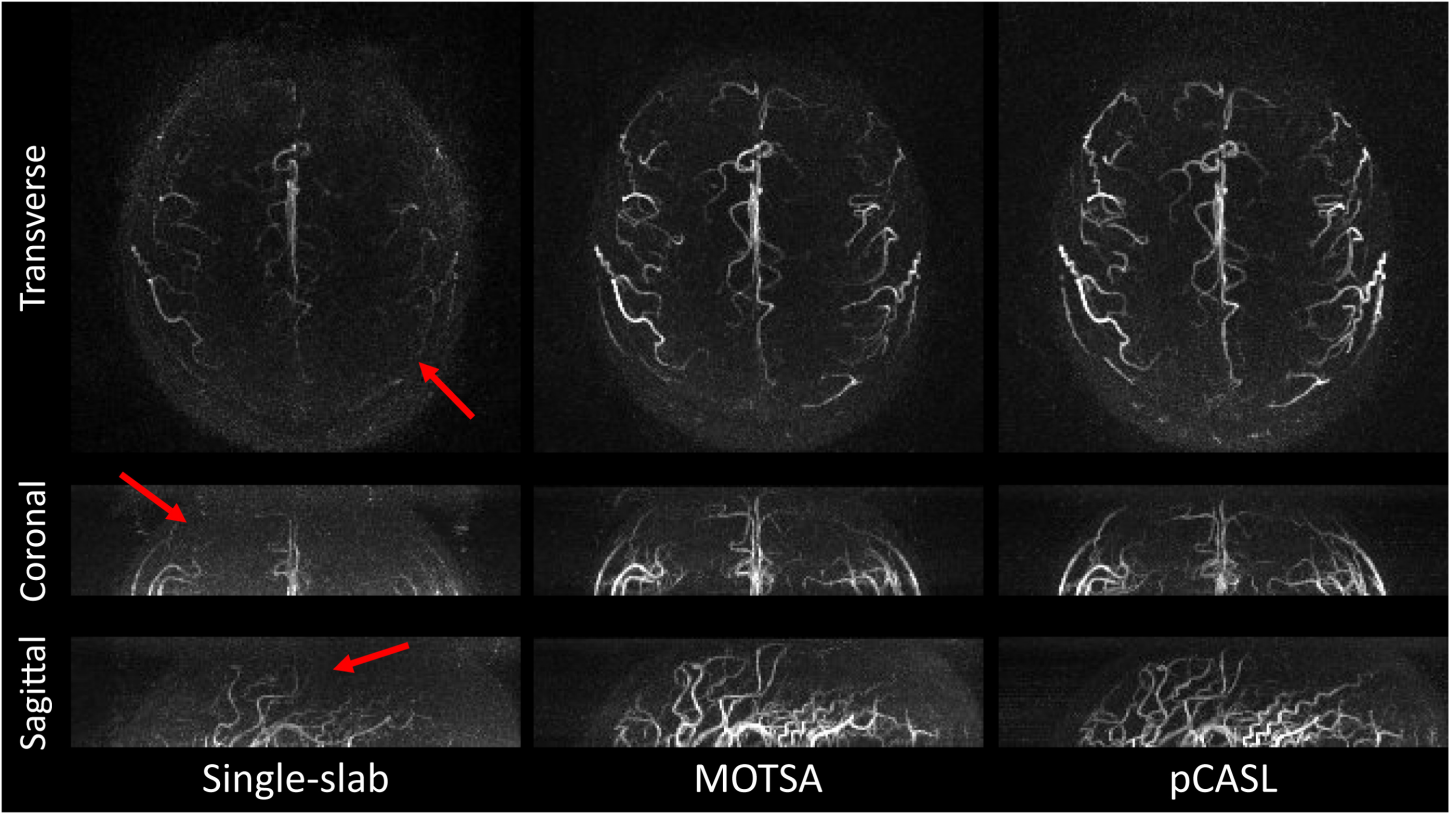
cMIP images comparing the third slab of MOTSA MRA, thin‐pCASL MRA, and the corresponding subregion from SS MRA in three orthogonal views. MOTSA MRA preserves distal arterial delineation comparable to thin‐pCASL MRA, whereas SS MRA exhibits reduced visibility of distal vessels (red arrows).

Figure 6 shows axial dynamic MIP and cMIP images comparing MOTSA MRA and pCASL MRA. Due to the longer labeling duration in pCASL, a substantial portion of labeled blood had already entered the imaging volume before data acquisition, resulting in predominant depiction of blood outflow dynamics. Although vessel delineation was comparable between the two techniques, depiction of complete inflow‐to‐outflow dynamics in pCASL MRA was less direct than in MOTSA MRA.

**FIGURE 6:**
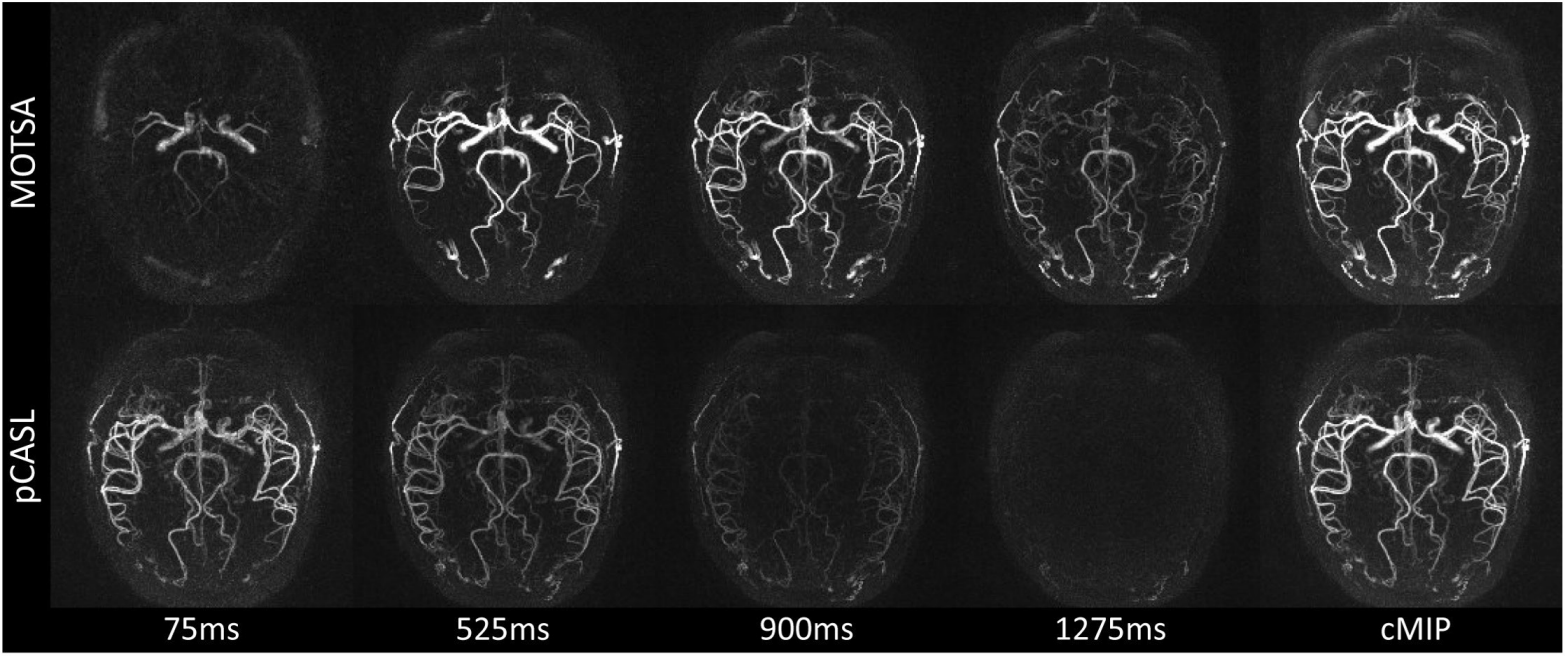
Representative axial MIP and cMIP images comparing MOTSA MRA and pCASL MRA. With long labeling duration, pCASL MRA predominantly depicts blood outflow dynamics, whereas MOTSA MRA captures complete arterial inflow‐to‐outflow bolus passage.

Pseudoinflow dynamics were derived from pCASL MRA with the post‐processing proposed in the previous work. An example of MOTSA MRA, pCASL MRA, and pseudoinflow of pCASL MRA is shown in Figure S3. Figure 7 compares dynamic blood flow curves from MOTSA MRA, pCASL MRA, and pseudoinflow of pCASL MRA across seven ROIs. Although overall temporal trends were similar, pCASL‐derived outflow and pseudoinflow curves exhibited flatter slopes and temporal offsets relative to MOTSA MRA, particularly in distal arterial segments (e.g., M2/3, M4, and P3/4). These discrepancies were more pronounced in regions with slower flow and longer arterial transit times. Yet, these differences are also expected given the fundamentally different labeling mechanisms, bolus characteristics, and temporal signal evolution of PASL‐ and pCASL‐based 4D MRA.

**FIGURE 7:**
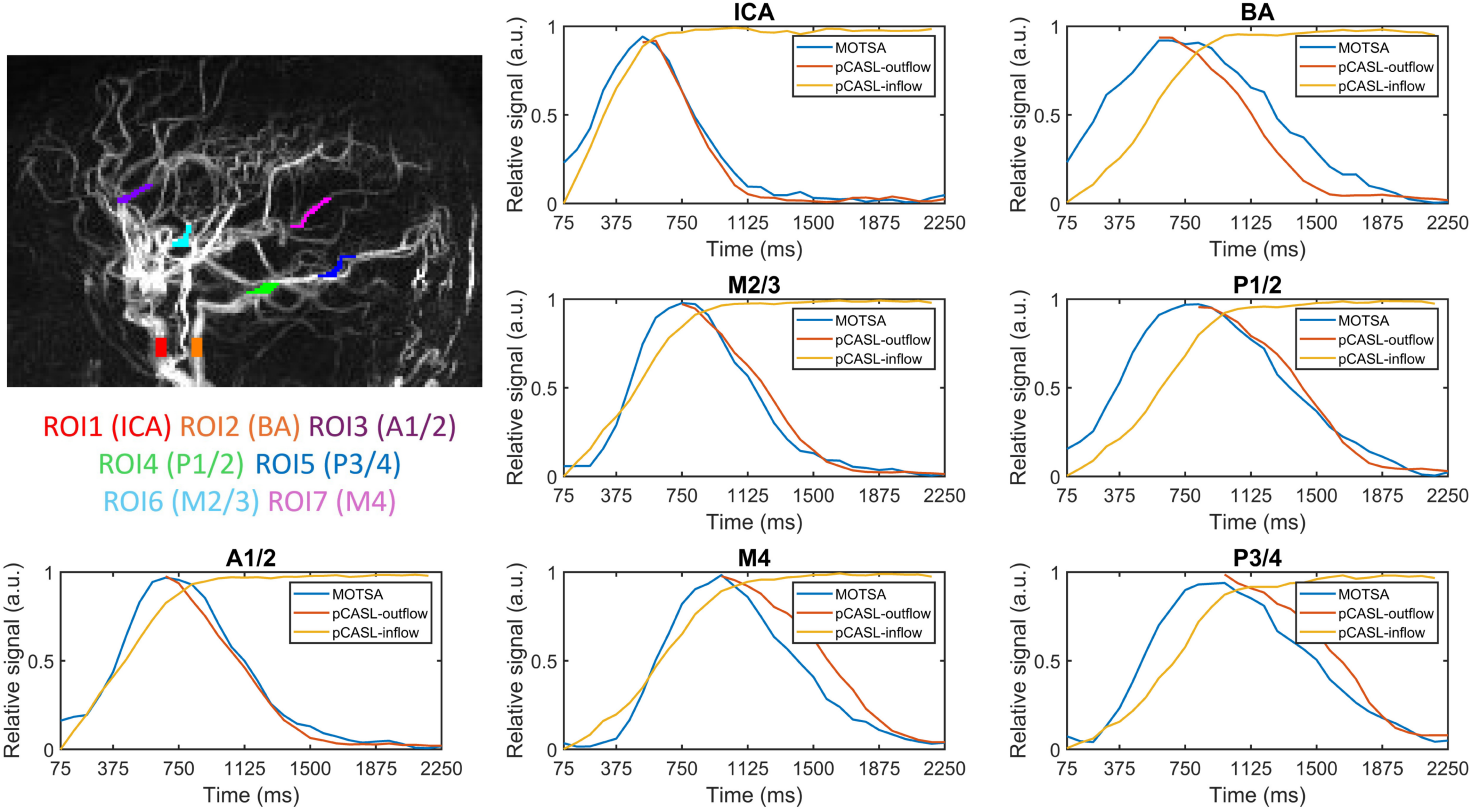
Dynamic blood signal curves measured in seven arterial ROIs for MOTSA MRA, pCASL MRA, and pseudoinflow of pCASL MRA. Although overall temporal trends are similar, outflow and pseudoinflow curves from pCASL MRA exhibit temporal offsets and flatter slopes in distal arterial segments.

Table 1a summarizes the averaged SNR, CNR, and SNR efficiency across three vessel levels for MOTSA MRA, SS MRA, and pCASL MRA using subspace low‐rank and NUFFT reconstruction, respectively. These quantitative results are further illustrated with the box plots shown in Figures S4 and S5. MOTSA MRA demonstrated SNR, CNR, and SNR efficiency values that were comparable to or higher than those of SS MRA and pCASL MRA across most vessel levels. Specifically, both MOTSA MRA and SS MRA exhibited high signal and contrast in Level 1 vessels (ICA and BA), which could be attributed to the enhancement in ICA and BA due to early blood inflow in PASL‐based MRA with minimal RF saturation. In contrast, SS MRA showed reduced signal and contrast in distal vessels (Level 3), whereas this reduction was less pronounced in MOTSA MRA and pCASL MRA. Together, these results indicate that MOTSA MRA maintained vessel signal and contrast across both proximal and distal vessel levels.

**TABLE 1:** Quantitative evaluation results of vessel delineation of (a) SNR, CNR, and SNR efficiency and (b) skeleton length in MOTSA MRA, SS MRA, and pCASL MRA.

(a)									
Vessel level	SNR			CNR			SNR efficiency		
	1	2	3	1	2	3	1	2	3
MOTSA MRA	90.86 ± 13.20	77.29 ± 9.55	52.13 ± 10.29	46.00 ± 7.24	39.92 ± 9.98	27.21 ± 8.37	4.32 ± 0.63	3.68 ± 0.45	2.48 ± 0.49
SS MRA	100.67 ± 22.03	69.99 ± 9.18	44.38 ± 6.62	52.49 ± 16.04	36.78 ± 9.39	23.35 ± 6.01	4.89 ± 1.07	3.40 ± 0.45	2.16 ± 0.32
pCASL MRA	82.68 ± 19.06	83.21 ± 17.23	65.54 ± 18.66	33.84 ± 8.62	34.18 ± 9.06	26.49 ± 7.11	3.35 ± 0.77	3.37 ± 0.70	2.66 ± 0.76
MOTSA MRA (NUFFT)	26.09 ± 7.64	17.63 ± 3.44	12.35 ± 3.43	13.66 ± 5.66	9.72 ± 6.48	6.53 ± 3.76	1.24 ± 0.36	0.84 ± 0.16	0.59 ± 0.16
SS MRA (NUFFT)	25.51 ± 12.07	14.80 ± 3.54	11.26 ± 3.20	12.97 ± 7.20	7.98 ± 4.89	5.98 ± 3.42	1.24 ± 0.59	0.72 ± 0.17	0.55 ± 0.16
pCASL MRA (NUFFT)	16.94 ± 6.68	18.29 ± 12.68	14.07 ± 5.65	7.73 ± 2.41	7.75 ± 3.12	6.26 ± 2.13	0.69 ± 0.27	0.74 ± 0.51	0.57 ± 0.23

(b)			
	MOTSA MRA	SS MRA	pCASL MRA
Skeleton length	3571.14 ± 627.88	2126.57 ± 302.69	3262.86 ± 447.19
Skeleton length (manual)	4528.86 ± 513.22	2504.50 ± 269.96	4180.93 ± 465.67

Quantitative vessel delineation results are summarized in Table 1b and illustrated in Figure S6. MOTSA MRA yielded a longer vessel skeleton length than SS MRA for both manual segmentation and neural‐network‐based analyses, indicating improved whole‐brain vessel delineation. Representative vessel skeleton MIP images using the neural network are shown in Figure 8. Because PASL‐ and pCASL‐based techniques exhibit fundamentally different blood dynamics, direct quantitative comparison between the two techniques should be interpreted cautiously.

**FIGURE 8:**
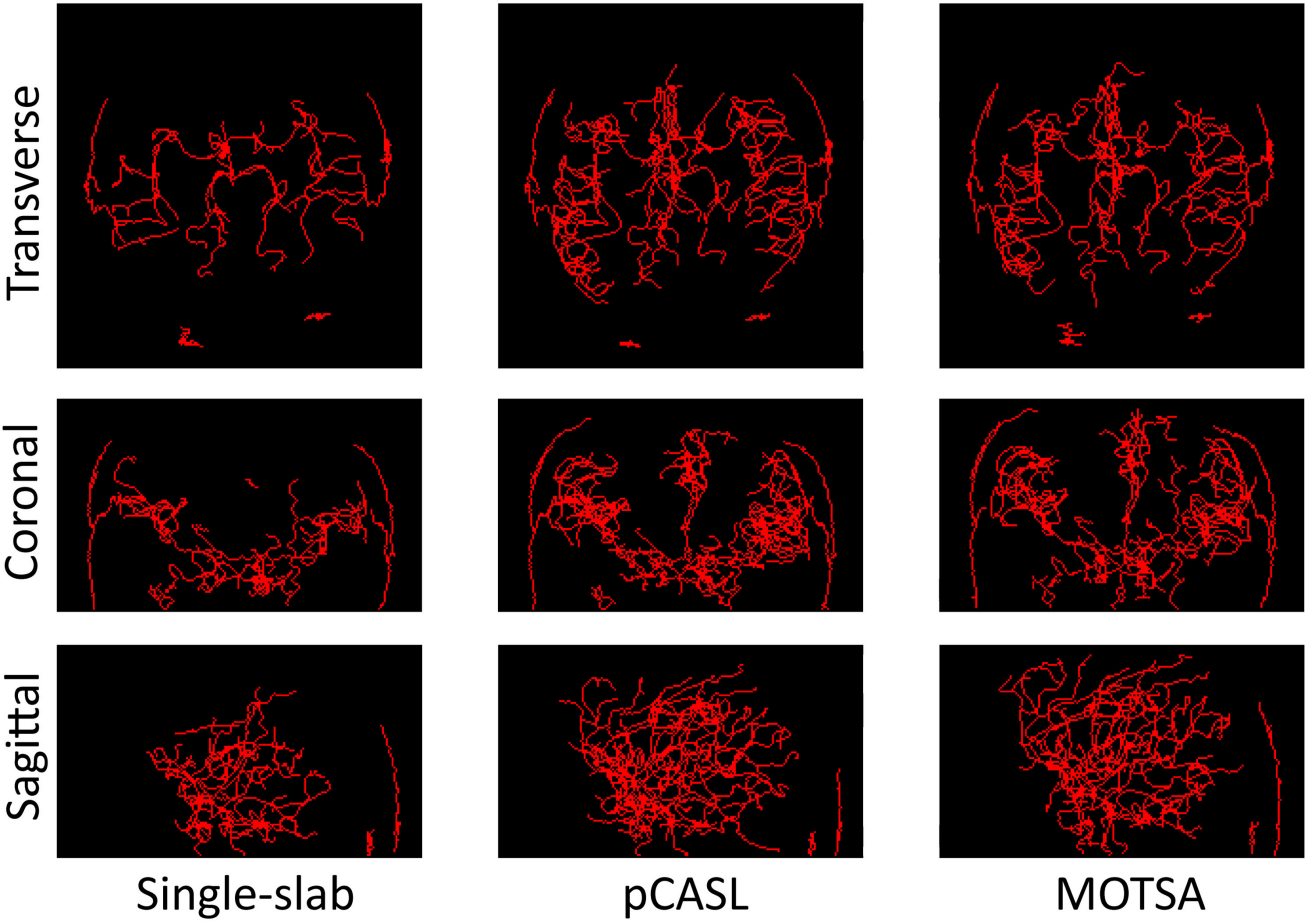
Representative vessel skeleton MIP images derived from automated vessel segmentation for MOTSA MRA, SS MRA, and pCASL MRA. MOTSA MRA demonstrates the best whole‐brain vessel skeleton.

Figure 9 presents selected dynamic MIP frames acquired with MOTSA MRA in transverse, coronal, and sagittal views. The images demonstrate continuous arterial inflow‐to‐outflow dynamics throughout the entire cerebral vasculature with high spatial and temporal resolution.

**FIGURE 9:**
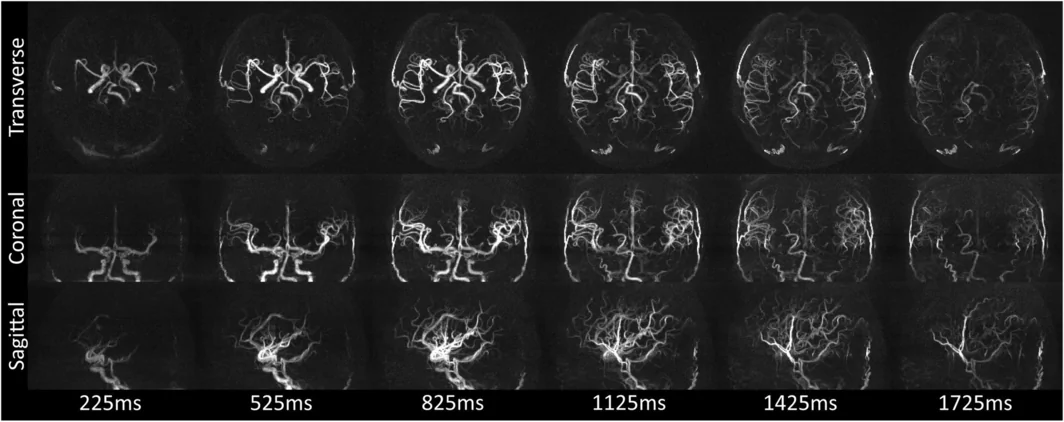
Selected dynamic MIP frames acquired using MOTSA MRA in transverse, coronal, and sagittal views. MOTSA MRA demonstrates continuous arterial inflow‐to‐outflow dynamics across the entire cerebral vasculature with high spatial and temporal resolution.

## 4 Discussion

In this work, we developed a practical solution to achieve a whole‐brain PASL‐based 4D MRA technique with improved delineation of distal small arteries while maintaining scan efficiency, which combined SOS golden‐angle radial sampling with an undersampled MOTSA. RF saturation effects on the inflow blood spins during the readout were significantly reduced through MOTSA, compared to a large single‐slab acquisition. Compared with conventional single‐slab 4D MRA, the proposed approach demonstrated preserved higher ASL signals when labeled blood flows along the arterial tree within the imaging volume, particularly in distal vessels. Comparisons with TOF‐MRA and pCASL‐based 4D MRA further highlight the ability of the proposed technique in comprehensive characterization of whole‐brain vasculature and blood flow with complete inflow‐to‐outflow dynamics.

To capture complete dynamic flow through the cerebral vasculature, PASL‐based 4D MRA commonly relies on extended look‐locker type readouts, which lead to cumulative RF saturation and progressive signal attenuation when labeled blood travels through the imaging volume. Although such saturation does not fundamentally alter temporal fidelity, it substantially degrades ASL signal and vessel visibility, especially in distal fine arterial segments. MOTSA was originally introduced in 3D TOF‐MRA to alleviate saturation effects on inflow spins and improve visualization of small vessels. Adapting this strategy to PASL‐based 4D MRA similarly reduces the total number of RF excitations experienced by labeled blood before entering each slab, thereby preserving inflow blood signal intensity and improving distal vessel delineation. This has been demonstrated through both visual inspection and quantitative comparisons between the proposed MOTSA and the conventional single‐slab PASL‐based 4D MRA, showing MOTSA MRA offers overall improved delineation of whole‐brain vasculature, especially the distal fine vasculature. Importantly, this approach enables improved spatial coverage without compromising temporal resolution or requiring a reduction in the number of RF excitations in the readout, thus maintaining the desired acquisition window length. Likewise, such a strategy could also be beneficial for ASL‐based conventional static or multiphase MRA with Cartesian sampling to achieve larger spatial coverage and better visualization of distal/slow flow, where a large number of k‐space segments or a prolonged Look‐Locker readout are used. Compared to 3D TOF‐MRA, ASL‐based 4D MRA inherently benefits from label/control subtraction, which effectively suppresses background tissue signal, and venous contamination is inherently reduced because only labeled arterial blood contributes to angiographic signals. In contrast, venous suppression in whole‐brain TOF‐MRA may become less effective when whole‐brain imaging volumes are required, potentially resulting in increased venous signal on MIP images. The impact of subject motion also differs between MOTSA and single‐slab acquisitions. Because k‐space data are acquired independently for each slab in MOTSA, subject motion may lead to interslab misregistration, signal discontinuities, or contrast mismatches, e.g., Venetian blind artifacts. In contrast, motion during single‐slab acquisition may affect a larger portion of the reconstructed volume, causing overall image blurring and degradation because entire k‐space data are acquired during the extended readout duration. Although Venetian blind artifacts were observed in some cases, similar to TOF‐MRA, which might affect the qualitative evaluation, these artifacts did not appear to substantially affect vessel continuity or quantitative evaluation reported in this study.

In addition to MOTSA, several other strategies may be used to mitigate RF saturation effects in ASL‐based angiographic imaging. Multislice 2D acquisition can substantially reduce RF saturation effects on inflowing spins to the best possible extent relative to volumetric imaging, thereby improving visualization of distal arteries, as demonstrated in previous work [37]. Furthermore, 2D imaging allows flexible expansion of spatial coverage. However, compared with 3D volumetric imaging, 2D acquisitions may involve trade‐offs in SNR and may offer limited opportunities for acceleration in image acquisition along kz direction, which could result in longer scan time. Ramped excitation, i.e., TONE, or variable flip angle (VFA) approaches [39, 40] applied spatially and temporally can be used to reduce signal saturation and improve visualization of distal vessels/late flow, both of which have also been applied in static or dynamic MRA techniques. However, the practical range available for VFA modulation is limited as the Ernst angle in spoiled GRE‐based ASL angiography is relatively small. Consequently, the improvement in saturation effect may thus be modest. Meanwhile, the application of VFA could further complicate the temporal signal evolution, potentially affecting quantitative analysis of arterial transit behavior or ASL signal modeling. RF saturation may also be mitigated through sequence parameter optimization, such as using smaller excitation FA, increasing echo spacing, or shortening the readout duration. However, PASL‐based 4D MRA typically requires a relatively long readout window to capture complete inflow‐to‐outflow dynamics, particularly in vascular territories with prolonged transit times or pathological conditions such as AVM and moyamoya disease. Although smaller flip angles and longer echo spacing can reduce cumulative RF saturation, these approaches introduce additional trade‐offs. Smaller FA may result in decreased vessel conspicuity due to compromised SNR, whereas longer echo spacing would reduce the possible temporal resolution, which might hamper the assessment of fast flow events (Figure S7). Collectively, within the imaging conditions investigated in this study, MOTSA provided an effective strategy and practical solution for mitigating RF saturation while maintaining a balance among temporal resolution, spatial coverage, and SNR. Yet, the relative benefit of MOTSA is expected to depend on the specific acquisition design and intended clinical or research application, and further optimization may be needed for different clinical applications.

In the current work, a STAR‐type PASL labeling scheme was employed, in which arterial blood spins inferior to the imaging volume were labeled. As an alternative, the flow‐sensitive alternating inversion recovery (FAIR)‐type labeling can also be used by employing the same inversion volume that fully covers all imaging slabs during slab‐selective inversion; however, for an extensive spatial coverage, such as whole‐brain imaging, the inversion profile of the slab‐selective IR may become imperfect, with a wider transition band that can adversely impact labeling efficiency. In contrast, the STAR labeling module can be implemented using a fixed inversion volume and relative distance from the imaging volume, facilitating consistent application across different slab configurations. However, one limitation of the STAR labeling scheme is that the off‐resonance labeling pulse may accidentally excite fat signal, potentially resulting in residual fat‐related artifacts, as observed in some cases in Figure 4. Additional fat suppression or water‐excitation techniques may help address these artifacts and could be incorporated in future implementations.

Apart from the pulsed ASL, pCASL‐based 4D MRA represents an alternative labeling strategy that can provide higher labeling efficiency and stronger blood signals given its longer labeling duration [27, 28, 31]. As a result, pCASL‐based 4D MRA yields good vessel delineation, including distal arteries. However, the long labeling duration leads to substantial filling of the arterial tree prior to image acquisition, resulting in predominant depiction of blood outflow dynamics, compared to pulsed ASL‐based 4D MRA. Although pseudoinflow information can be generated through image‐based subtraction approaches [32], the resulting dynamics are potentially sensitive to noise and susceptible to artifacts arising from the different sampling patterns from frame to frame when non‐Cartesian acquisition is applied. Moreover, such pseudoinflow information may complicate the interpretation, particularly in distal vessels with slow flow and prolonged arterial transit times, where a mixture of inflow and outflow dynamics could happen simultaneously. These effects were evident in the present study, in which pseudoinflow curves derived from pCASL‐based 4D MRA exhibited temporal offsets and flattened slopes relative to the directly measured inflow dynamics obtained with the proposed technique. Applying postlabeling delay or a longer labeling duration may partially alleviate this effect (Figure S8) but comes at the expense of longer scan times and potentially insufficient correction for the most distal vascular territories. Importantly, these differences are expected given the fundamentally different labeling mechanisms and temporal behavior of labeled blood in PASL‐ and pCASL‐based 4D MRA and therefore should not be interpreted as evidence of inaccuracy in either ASL technique.

Several limitations of this study should be noted. First, imaging parameters, e.g., number of fully sampled central partitions, total number of TRs, were not perfectly matched between the proposed MOTSA MRA and the conventional single‐slab 4D MRA due to protocol constraints, which resulted in slight differences in effective kz acceleration rate (1.75 for MOTSA acquisition and 1.57 for single‐slab acquisition) and may influence SNR efficiency comparisons. Nevertheless, despite increased undersampling, the proposed method consistently demonstrated comparable or improved SNR, CNR, and vessel delineation metrics. Further protocol optimization will be needed to enhance the scan efficiency to fit a specific clinical need. Second, image evaluation focused primarily on vessel delineation and contrast. A more comprehensive assessment of image quality in terms of clinical diagnostic ability may be warranted in future work. Thirdly, the neural network used for vessel segmentation is originally trained on PASL‐based 4D MRA datasets, which may introduce bias when applied to pCASL‐based MRA data. To reduce this effect, a 3D spatial‐only segmentation network was used, and no retraining or fine‐tuning was performed for the current datasets. Nevertheless, manual vessel segmentation performed by two independent raters demonstrated findings consistent with the neural‐network‐based analysis, supporting the robustness of the vessel delineation results. Additionally, identical reconstruction regularization parameters were applied across all 4D MRA methods to maintain comparable reconstruction behavior, noise characteristics, temporal fidelity, and image appearance, which may not represent the optimal configuration for each technique. Although individually optimized regularization settings could potentially yield different performance trade‐offs for each technique, quantitative image quality trends remained largely consistent between low‐rank subspace reconstruction and NUFFT reconstruction, supporting the reported findings. Furthermore, pCASL‐based MRA may exhibit less effective background suppression during early temporal frames because of the longer interval between presaturation and image acquisition, which could also potentially influence its performance. Direct quantitative comparison between PASL‐ and pCASL‐based MRA should thus be cautious. Finally, this study was conducted in a small cohort of healthy volunteers, and further validation in patients with cerebrovascular disease is needed.

In conclusion, this study introduces a whole‐brain ASL‐based 4D MRA technique that integrates SOS golden‐angle radial sampling with the MOTSA to reduce RF saturation effects and improve distal vessel delineation. The proposed approach enables time‐resolved visualization of complete arterial inflow‐to‐outflow dynamics with high spatiotemporal resolution and efficiency, offering a robust framework for noncontrast‐enhanced cerebrovascular imaging.

## Author Contributions

**Zhao T. and Yan L.:** conceptualization, methodology, and experimental design. **Zhao T, Chung SH, He Y., Huang Z., Tang J., Yan L.:** data collection, image reconstruction and processing, and data analysis. **Zhao T., Chung S.H., He Y., Huang Z., Tang J., Ansari S.A., Yan L.:** result interpretation. **Zhao T., Chung S.H., Ansari S.A., Yan L.:** manuscript writing. **Zhao T., Chuang S.H., He Y., Yan L.:** manuscript revision.

## Funding

This work was supported by the National Institute of Neurological Disorders and Stroke (R01NS118019, RF1NS139370), National Institute on Aging (R01AG072490), BrightFocus Foundation (A20201411S).

## Conflicts of Interest

The authors declare no conflicts of interest.

## Supporting information


**Table S1:** Signal saturation factor across arterial segments of SS MRA to MOTSA MRA.
**Figure S1:** Example of image stitching in MOTSA MRA. Overlapped slices between adjacent slabs are average combined (seven edge slices from 48‐slice slab with slab distance factor of −15%) to form a single slab.
**Figure S2:** Example of sagittal MIP images comparing thin‐slab pCASL to single large slab pCASL with the corresponding region. Thin‐slab pCASL presents better distal delineation because of minimal signal decay and RF saturation, primarily due to thin‐slab acquisition and smaller gap from labeling plane to imaging plane.
**Figure S3:** Representative axial MIP images comparing MOTSA MRA, pCASL MRA, and pseudoinflow of pCASL MRA. Similar early‐phase inflow patterns are observed between MOTSA MRA and pseudoinflow of pCASL MRA; however, vessel discontinuities and streaking artifacts are evident in the pseudoinflow of pCASL MRA.
**Figure S4:** Box plots of (a) SNR, (b) CNR, and (c) SNR efficiency comparing MOTSA MRA, SS MRA, and pCASL MRA across three vessel levels with subspace low‐rank reconstruction. MOTSA MRA demonstrates comparable to higher SNR, CNR, and SNR efficiency across all vessel levels.
**Figure S5:** Box plots of (a) SNR, (b) CNR, and (c) SNR efficiency comparing MOTSA MRA, SS MRA, and pCASL MRA across three vessel levels using NUFFT reconstruction. MOTSA MRA demonstrates comparable to higher SNR, CNR, and SNR efficiency across all vessel levels.
**Figure S6:** (a) Box plots of skeleton length comparing MOTSA MRA, SS MRA, and pCASL MRA with manual segmentation and neural network. MOTSA MRA shows comparable to longer total vessel delineation. (b) Inter‐rater reproducibility of manual segmentation with two raters. Excellent interrater agreement is achieved with ICC = 0.98.
**Figure S7:** Coronal MIP images comparing MOTSA MRA with single‐slab MRA with different parameter settings. Both the small flip angle and longer echo spacing can partially alleviate RF saturation to a certain extent, yet cannot outperform the MOTSA MRA (blue arrow) in the distal vessel delineations. In addition, the small flip angle has a compromised signal‐to‐noise ratio, whereas the longer echo spacing may suffer from signal dephasing induced by turbulent flow due to longer TE (red arrow) and reduced temporal resolution.
**Figure S8:** Dynamic blood signal curves measured in three distal arterial ROIs for MOTSA MRA, pCASL MRA, and pseudoinflow of pCASL MRA using a labeling duration of 2 s. Improved agreement with MOTSA MRA is observed in the M2/3 and M4 segments, whereas discrepancies persist in the P3/4 segments.

## Data Availability

The data that support the findings of this study are available on request from the corresponding author. The data are not publicly available due to privacy or ethical restrictions.

## References

[1] M. L. Portegies , P. J. Koudstaal , and M. A. Ikram , “Cerebrovascular Disease,” in Handbook of Clinical Neurology, vol. 138 (Elsevier, 2016), 239–261, 10.1016/B978-0-12-802973-2.00014-8. 27637962

[2] M. J. Donahue , E. Achten , P. M. Cogswell , et al., “Consensus Statement on Current and Emerging Methods for the Diagnosis and Evaluation of Cerebrovascular Disease,” Journal of Cerebral Blood Flow and Metabolism 38, no. 9 (2018): 1391–1417, 10.1177/0271678X17721830. PMC6125970 28816594

[3] F. Liu , Y. Yao , B. Zhu , Y. Yu , R. Ren , and Y. Hu , “The Novel Imaging Methods in Diagnosis and Assessment of Cerebrovascular Diseases: An Overview,” Frontiers in Medicine 11 (2024): 1269742, 10.3389/fmed.2024.1269742. PMC11039813 38660416

[4] S. Shaban , B. Huasen , A. Haridas , et al., “Digital Subtraction Angiography in Cerebrovascular Disease: Current Practice and Perspectives on Diagnosis, Acute Treatment and Prognosis,” Acta Neurologica Belgica 122, no. 3 (2022): 763–780, 10.1007/s13760-021-01805-z. 34553337

[5] M. E. MacDonald and R. Frayne , “Cerebrovascular MRI: A Review of State‐of‐the‐Art Approaches, Methods and Techniques,” NMR in Biomedicine 28, no. 7 (2015): 767–791, 10.1002/nbm.3322. 26010775

[6] I. Koktzoglou , O. Ozturk , M. T. Walker , et al., “Quantitative Time‐of‐Flight Head Magnetic Resonance Angiography of Cerebrovascular Disease,” Journal of Magnetic Resonance Imaging 61, no. 1 (2025): 404–412, 10.1002/jmri.29395. PMC11502504 38662966

[7] A. Sakata , Y. Fushimi , T. Okada , et al., “Evaluation of Cerebral Arteriovenous Shunts: A Comparison of Parallel Imaging Time‐of‐Flight Magnetic Resonance Angiography (TOF‐MRA) and Compressed Sensing TOF‐MRA to Digital Subtraction Angiography,” Neuroradiology 63, no. 6 (2021): 879–887, 10.1007/s00234-020-02581-y. 33063222

[8] A. Conger , C. Kulwin , M. T. Lawton , and A. A. Cohen‐Gadol , “Diagnosis and Evaluation of Intracranial Arteriovenous Malformations,” Surgical Neurology International 6 (2015): 76, 10.4103/2152-7806.156866. PMC4429335 25984390

[9] J. P. Mohr , J. Kejda‐Scharler , and J. Pile‐Spellman , “Diagnosis and Treatment of Arteriovenous Malformations,” Current Neurology and Neuroscience Reports 13, no. 2 (2013): 324, 10.1007/s11910-012-0324-1. 23307509

[10] T. J. Vachharajani , “Diagnosis of Arteriovenous Fistula Dysfunction,” Seminars in Dialysis 25, no. 4 (2012): 445–450, 10.1111/j.1525-139X.2012.01094.x. 22694731

[11] A. A. Al‐Jaishi , A. R. Liu , C. E. Lok , J. C. Zhang , and L. M. Moist , “Complications of the Arteriovenous Fistula: A Systematic Review,” Journal of the American Society of Nephrology 28, no. 6 (2017): 1839–1850, 10.1681/ASN.2016040412. PMC5461784 28031406

[12] J. Gutierrez , T. N. Turan , B. L. Hoh , and M. I. Chimowitz , “Intracranial Atherosclerotic Stenosis: Risk Factors, Diagnosis, and Treatment,” Lancet Neurology 21, no. 4 (2022): 355–368, 10.1016/S1474-4422(21)00376-8. 35143758

[13] M. Iv , O. Choudhri , R. L. Dodd , et al., “High‐Resolution 3D Volumetric Contrast‐Enhanced MR Angiography With a Blood Pool Agent (Ferumoxytol) for Diagnostic Evaluation of Pediatric Brain Arteriovenous Malformations,” Journal of Neurosurgery. Pediatrics 22, no. 3 (2018): 251–260, 10.3171/2018.3.PEDS17723. 29882734

[14] S. J. Riederer , C. R. Haider , E. A. Borisch , P. T. Weavers , and P. M. Young , “Recent Advances in 3D Time‐Resolved Contrast‐Enhanced MR Angiography,” Journal of Magnetic Resonance Imaging 42, no. 1 (2015): 3–22, 10.1002/jmri.24880. PMC4478169 26032598

[15] X. Golay , J. Hendrikse , and T. C. Lim , “Perfusion Imaging Using Arterial Spin Labeling,” Topics in Magnetic Resonance Imaging 15, no. 1 (2004): 10–27, 10.1097/00002142-200402000-00003. 15057170

[16] J. A. Detre , J. S. Leigh , D. S. Williams , and A. P. Koretsky , “Perfusion Imaging,” Magnetic Resonance in Medicine 23, no. 1 (1992): 37–45, 10.1002/mrm.1910230106. 1734182

[17] L. Hernandez‐Garcia , V. Aramendia‐Vidaurreta , D. S. Bolar , et al., “Recent Technical Developments in ASL: A Review of the State of the Art,” Magnetic Resonance in Medicine 88, no. 5 (2022): 2021–2042, 10.1002/mrm.29381. PMC9420802 35983963

[18] Y. Suzuki , N. Fujima , and M. J. P. van Osch , “Intracranial 3D and 4D MR Angiography Using Arterial Spin Labeling: Technical Considerations,” Magnetic Resonance in Medical Sciences 19, no. 4 (2020): 294–309, 10.2463/mrms.rev.2019-0096. PMC7809141 31761840

[19] X. Bi , P. Weale , P. Schmitt , S. Zuehlsdorff , and R. Jerecic , “Non‐contrast‐Enhanced Four‐Dimensional (4D) Intracranial MR Angiography: A Feasibility Study,” Magnetic Resonance in Medicine 63, no. 3 (2010): 835–841, 10.1002/mrm.22220. 20187191

[20] L. Yan , N. Salamon , and D. J. Wang , “Time‐Resolved Noncontrast Enhanced 4‐D Dynamic Magnetic Resonance Angiography Using Multibolus TrueFISP‐Based Spin Tagging With Alternating Radiofrequency (TrueSTAR),” Magnetic Resonance in Medicine 71, no. 2 (2014): 551–560, 10.1002/mrm.24689. PMC3675183 23440649

[21] L. Yan , S. Wang , Y. Zhuo , et al., “Unenhanced Dynamic MR Angiography: High Spatial and Temporal Resolution by Using True FISP‐Based Spin Tagging With Alternating Radiofrequency,” Radiology 256, no. 1 (2010): 270–279, 10.1148/radiol.10091543. PMC2897689 20574100

[22] Z. Zhou , F. Han , S. Yu , et al., “Accelerated Noncontrast‐Enhanced 4‐Dimensional Intracranial MR Angiography Using Golden‐Angle Stack‐of‐Stars Trajectory and Compressed Sensing With Magnitude Subtraction,” Magnetic Resonance in Medicine 79, no. 2 (2018): 867–878, 10.1002/mrm.26747. PMC5675831 28480537

[23] T. Zhao , J. Tang , C. Krumpelman , et al., “Highly Accelerated Non‐Contrast‐Enhanced Time‐Resolved 4D MRA Using Stack‐of‐Stars Golden‐Angle Radial Acquisition With a Self‐Calibrated Low‐Rank Subspace Reconstruction,” Magnetic Resonance in Medicine 93, no. 2 (2025): 615–629, 10.1002/mrm.30304. PMC11604851 39344291

[24] O. Togao , A. Hiwatashi , M. Obara , et al., “4D ASL‐Based MR Angiography for Visualization of Distal Arteries and Leptomeningeal Collateral Vessels in Moyamoya Disease: A Comparison of Techniques,” European Radiology 28, no. 11 (2018): 4871–4881, 10.1007/s00330-018-5462-7. 29737389

[25] J. Jang , P. Schmitt , B. Y. Kim , et al., “Non‐contrast‐Enhanced 4D MR Angiography With STAR Spin Labeling and Variable Flip Angle Sampling: A Feasibility Study for the Assessment of Dural Arteriovenous Fistula,” Neuroradiology 56, no. 4 (2014): 305–314, 10.1007/s00234-014-1336-0. 24526141

[26] J. Xu , D. Shi , C. Chen , et al., “Noncontrast‐Enhanced Four‐Dimensional MR Angiography for the Evaluation of Cerebral Arteriovenous Malformation: A Preliminary Trial,” Journal of Magnetic Resonance Imaging 34, no. 5 (2011): 1199–1205, 10.1002/jmri.22699. 21769984

[27] H. Wu , W. F. Block , P. A. Turski , C. A. Mistretta , and K. M. Johnson , “Noncontrast‐Enhanced Three‐Dimensional (3D) Intracranial MR Angiography Using Pseudocontinuous Arterial Spin Labeling and Accelerated 3D Radial Acquisition,” Magnetic Resonance in Medicine 69, no. 3 (2013): 708–715, 10.1002/mrm.24298. PMC3424331 22532423

[28] H. Wu , W. F. Block , P. A. Turski , et al., “Noncontrast Dynamic 3D Intracranial MR Angiography Using Pseudo‐Continuous Arterial Spin Labeling (PCASL) and Accelerated 3D Radial Acquisition,” Journal of Magnetic Resonance Imaging 39, no. 5 (2014): 1320–1326, 10.1002/jmri.24279. PMC3984365 24129947

[29] L. Feng , “Golden‐Angle Radial MRI: Basics, Advances, and Applications,” Journal of Magnetic Resonance Imaging 56, no. 1 (2022): 45–62, 10.1002/jmri.28187. PMC9189059 35396897

[30] M. A. Griswold , P. M. Jakob , R. M. Heidemann , et al., “Generalized Autocalibrating Partially Parallel Acquisitions (GRAPPA),” Magnetic Resonance in Medicine 47, no. 6 (2002): 1202–1210, 10.1002/mrm.10171. 12111967

[31] L. Feng , “4D Golden‐Angle Radial MRI at Subsecond Temporal Resolution.” NMR in Biomedicine 36, no. 2 (2023): e4844, 10.1002/nbm.4844. PMC9845193 36259951

[32] D. Kopeinigg and R. Bammer , “Time‐Resolved Angiography Using Inflow Subtraction (TRAILS),” Magnetic Resonance in Medicine 72, no. 3 (2014): 669–678, 10.1002/mrm.24985. PMC4396866 24166577

[33] J. A. Fessler and B. P. Sutton , “Nonuniform Fast Fourier Transforms Using Min‐Max Interpolation,” IEEE Transactions on Signal Processing 51, no. 2 (2003): 560–574, 10.1109/Tsp.2002.807005.

[34] S. H. Chung , Z. Wang , T. Zhao , et al., “ASL 4D MRA Intracranial Vessel Segmentation With Deep Learning U‐Nets,” Magnetic Resonance in Medicine 95 (2025): 2384–2396, 10.1002/mrm.70173. PMC12850589 41207868

[35] J. M. Bland and D. G. Altman , “Measuring Agreement in Method Comparison Studies,” Statistical Methods in Medical Research 8, no. 2 (1999): 135–160, 10.1177/096228029900800204. 10501650

[36] J. J. Bartko , “The Intraclass Correlation Coefficient as a Measure of Reliability,” Psychological Reports 19, no. 1 (1966): 3–11, 10.2466/pr0.1966.19.1.3. 5942109

[37] Y. Suzuki , I. Koktzoglou , Z. Li , P. Jezzard , and T. Okell , “Improved Visualization of Intracranial Distal Arteries With Multiple 2D Slice Dynamic ASL‐MRA and Super‐Resolution Convolutional Neural Network,” Magnetic Resonance in Medicine 92, no. 6 (2024): 2491–2505, 10.1002/mrm.30245. 39155401

[38] D. Atkinson , M. Brant‐Zawadzki , G. Gillan , D. Purdy , and G. Laub , “Improved MR Angiography: Magnetization Transfer Suppression With Variable Flip Angle Excitation and Increased Resolution,” Radiology 190, no. 3 (1994): 890–894, 10.1148/radiology.190.3.8115646. 8115646

[39] T. Nagele , U. Klose , W. Grodd , D. Petersen , and J. Tintera , “The Effects of Linearly Increasing Flip Angles on 3D Inflow MR Angiography,” Magnetic Resonance in Medicine 31, no. 5 (1994): 561–566, 10.1002/mrm.1910310515. 8015412

[40] M. Wang , Y. Ma , F. Chen , F. Zhou , J. Zhang , and B. Zhang , “Acceleration of pCASL‐Based Cerebral 4D MR Angiography Using Compressed SENSE: A Comparison With SENSE,” Frontiers in Neurology 13 (2022): 796271, 10.3389/fneur.2022.796271. PMC8977489 35386411

