# Supplementary material for: Whole‐Brain 4D MRA With Improved Vessel Delineation Using Undersampled Multiple Overlapping Thin‐Slab Radial Acquisition
Source: NMR Biomed. 2026 Oct 22;39(12):e70426. doi: 10.1002/nbm.70426 (PMC13688705; doi:10.1002/nbm.70426)
Supplement: Supplementary file 1 [file NBM-39-e70426-s001.docx]

Supplementary Table S1. Signal saturation factor across arterial segments of SS MRA to MOTSA MRA.

|  | **ICA (reference)** | **BA** | **P1/2** | **P3/4** | **ACA** | **M2/3** | **M4** |
| --- | --- | --- | --- | --- | --- | --- | --- |
| Signal saturation factor | - | 0.963 | 0.944 | 0.781 | 0.716 | 0.712 | 0.694 |

Supplementary Figure S1. Example of image stitching in MOTSA MRA. Overlapped slices between adjacent slabs are average combined (7 edge slices from 48-slice slab with slab distance factor of -15%) to form a single slab.

Supplementary Figure S2. Example of sagittal MIP images comparing thin-slab pCASL to single large slab pCASL with the corresponding region. Thin-slab pCASL presents better distal delineation because of minimal signal decay and RF saturation, primarily due to thin-slab acquisition and smaller gap from labeling plane to imaging plane.

Supplementary Figure S3. Representative axial MIP images comparing MOTSA MRA, pCASL MRA, and pseudo-inflow of pCASL MRA. Similar early-phase inflow patterns are observed between MOTSA MRA and pseudo-inflow of pCASL MRA; however, vessel discontinuities and streaking artifacts are evident in the pseudo-inflow of pCASL MRA.

Supplementary Figure S4. Box plots of (a) SNR, (b) CNR, and (c) SNR efficiency comparing MOTSA MRA, SS MRA, and pCASL MRA across three vessel levels with subspace low-rank reconstruction. MOTSA MRA demonstrates comparable to higher SNR, CNR, and SNR efficiency across all vessel levels.

Supplementary Figure S5. Box plots of (a) SNR, (b) CNR, and (c) SNR efficiency comparing MOTSA MRA, SS MRA, and pCASL MRA across three vessel levels using NUFFT reconstruction. MOTSA MRA demonstrates comparable to higher SNR, CNR, and SNR efficiency across all vessel levels.

Supplementary Figure S6. (a) Box plots of skeleton length comparing MOTSA MRA, SS MRA, and pCASL MRA with manual segmentation and neural network. MOTSA MRA shows comparable to longer total vessel delineation. (b) inter-rater reproducibility of manual segmentation with two raters. Excellent inter-rater agreement is achieved with ICC = 0.98.

Supplementary Figure S7. Coronal MIP images comparing MOTSA MRA with single slab MRA with different parameter settings. Both small flip angle and longer echo spacing can partially alleviate RF saturation to a certain extent, yet cannot outperform the MOTSA MRA (blue arrow) in the distal vessel delineations. In addition, the small flip angle has compromised signal to noise ratio, while the longer echo spacing may suffer from signal dephasing induced by turbulent flow due to longer TE (red arrow) and reduced temporal resolution.

Supplementary Figure S8. Dynamic blood signal curves measured in three distal arterial ROIs for MOTSA MRA, pCASL MRA, and pseudo-inflow of pCASL MRA using a labeling duration of 2 s. Improved agreement with MOTSA MRA is observed in the M2/3 and M4 segments, whereas discrepancies persist in the P3/4 segments.
